# *Mycobacterium tuberculosis* Induces Expansion of Foxp3 Positive CD4 T-cells with a Regulatory Profile in Tuberculin Non-sensitized Healthy Subjects: Implications for Effective Immunization against TB

**DOI:** 10.4172/2155-9899.1000428

**Published:** 2016-06-16

**Authors:** Christina S Hirsch, Roxana Rojas, Mianda Wu, Zahra Toossi

**Affiliations:** 1 Division of Infectious Diseases, Case Western Reserve University, Cleveland OH, USA; 2 Veterans Affairs Medical Center, Cleveland, OH, USA

**Keywords:** Tuberculosis, TGFβ, TLR2, IDO, T-reg, IL-6, IL-8

## Abstract

**Objective:**

Infection by MTB or exposure to MTB constituents is associated with intense microbial stimulation of the immune system, through both antigenic and TLR components, and induction of a milieu that is rich in pro-inflammatory/anti-inflammatory cytokines. Here, we addressed the basis of induced regulatory T-cell (iT-reg) expansion in response to MTB stimulation, in the absence of prior T cell antigen responsiveness.

**Methods:**

PBMC from HIV-1 un-infected TST negative and TST positive control subjects were stimulated by virulent MTB H37Rv lysate (L), a French press preparation of MTB that includes all bacterial components. Phenotype of MTB H37RvL induced iT-reg was assessed using immunostaining and flow cytometry. Functional capacity of iT-reg was assessed using ^3^H-Thymidine incorporation and IFNγ production of non-adherent T cells (NAC) in the presence or absence of iT-reg in corresponding culture supernatants in response to TCR stimulation. Realtime PCR was used to assess IDO and FoxP3 mRNA expression.

**Results:**

The capacity of MTB H37RvL to induce CD4^+^CD25^hi+^ Foxp3^+^ T-cells in PBMC from TST negative subjects was robust (p<0.001), and in fact comparable to induction of iT-reg in PBMC from TST positive subjects. MTB-induced CD4^+^CD25^hi+^ T-reg were TGFβ positive (p<0.05). Further, MTB H37RvL induced CD4^+^CD25^hi+^ Foxp3^+^ iT-reg suppressed ^3^H-Thymidine incorporation and IFNγ production of non-adherent T cells (NAC) in response to TCR stimulation. MTB H37RvL induction of iT-reg was significantly stronger (p<0.01) than that by TLR-2, TLR-4, TLR-9 ligands, or combination of all TLR ligands. MTB H37RvL inducted indoleamine 2,3-dideoxygenase (IDO) mRNA expression in monocytes (p<0.001), and co-culture with the IDO inhibitor, D-1MT, decreased frequencies of T-reg (p<0.05). Inhibition of TGFβ by siRNA reduced Foxp3 mRNA expression in CD4 T cells (p<0.05).

**Conclusion:**

Therefore, MTB and its components expand functional iT-reg in human mononuclear cells from MTB non-sensitized subjects. Also, MTB-induced iT-reg expansion depends on mononuclear phagocyte expression of both TGFβ and IDO.

## Introduction

Expansion of Foxp3+CD4 T cells with regulatory function (T-reg) associated with infections and malignancies have been shown to be primarily due to T cell immune responses to microbial or tumor antigens. Bystander inflammation, concomitant with immune responses, also conditions naïve T cells to Foxp3 expression and reduced effector T cell differentiation [1]. A role for induced (i) T-reg in limiting immunogenicity of novel antigens subsequent to immunization or infection in humans has been examined, however, remains controversial [2,3].

Mononuclear cells recruited to sites of *M. tuberculosis* (MTB) infection or novel MTB antigens, are exposed to MTB Toll-like receptor (TLR) ligands. MTB is rich in TLR2 ligands [4,5], and a role for TLR2 ligand in expansion of T-reg has been previously shown [6]. However, TLR2 ligation leads to reduction in the suppressive function of T-reg also [7]. The role of TLR2 and other TLR ligands of MTB in accumulation of iT-reg have not been fully examined.

At sites of MTB infection, recruited mononuclear cells are also exposed to an intense TH1 response in a milieu high in immune activation [8]. In this latter study, Foxp3 mRNA expression in pleural fluid mononuclear cells correlated with local levels of IL-6 and IL-8 and to a lesser extent TGFβ, but not at all with levels of IFNγ. These data imply support of Foxp3 mRNA expression in mononuclear cells by the intense inflammation *in situ*, rather than secondary to the developing TH1 responses against MTB. The role of inflammatory cytokines in promotion of Foxp3 expression in response to MTB antigens is not clear.

In the absence of immune cytokines, expansion of functional iT-reg is dependent on contact with monocyte derived dendritic cells (DC) [9]. Indoleamine 2,3-dideoxygenase (IDO) expressed by DC promotes iT-reg development, and additionally blocks conversion of T-reg to TH17-like cells [10]. The role of IDO in expansion of iT reg in human mononuclear phagocytes in response to MTB antigens has not been studied.

Here, we examined the capacity of MTB to expand iT-reg in the absence of established T-cell immune responses to the pathogen. For this purpose, PBMC from subjects who did not have evidence of prior exposure to MTB (i.e. Tuberculin skin test [TST] negative individuals) were examined. The molecular and cellular basis and the functional phenotype of MTB-induced T-reg were assessed. MTB H37Rvlysate (L) induced expansion of CD4^+^CD25^hi+^Foxp3^+^ T-cells that were characterized by increased intracellular expression of TGFβ, and suppression of TCR stimulated T-cell responses. Monocyte expression of both IDO and TGFβ were found to be involved in expansion of iT-reg by MTB H37RvL.

## Methods

### Study subjects

Healthy tuberculin skin test negative and positive volunteers were recruited among the research community at Case Western Reserve University in Cleveland, Ohio. Informed consent was obtained from all enrollees prior to participation in this study.

To ascertain lack of T-cell responses to MTB as a result of *in vitro* ‘sensitization’ to MTB antigens in TST negative subjects as suggested before [11], standard proliferation assays to MTB H37Rv lysate (L) were performed on all donors. No significant proliferation in response to MTB H37RvL (stimulation index ≤ 2) was observed in the TST negative subjects recruited.

### Reagents

Whole cell lysate of MTB H37Rv (MTB H37RvL) [Tuberculosis Research Materials and Vaccine Testing Contract (NO1-AI-75320)], a crude French press preparation of gamma-irradiated virulent MTB grown to log phase was used. This preparation includes all MTB proteins, lipids, and carbohydrates. LPS contamination of this preparation as assessed by Limulus Lysate assay (ThermoFisher, Waltham, MA) was negligible. The TLR agonists Pam-3-cysk4 (TLR-2 ligand) (EMC Micro-collections, Tuebingen, Germany), LPS (TLR-4 ligand) (Sigma Fine Chemicals) and CPG (TLR-9 ligand) (Coley Pharmaceuticals, Wellesley, MA) were purchased. The selective IDO inhibitor, D-1-methyl-tryptopahn (D-1MT) (Sigma Fine Chemicals) was used at 100 μmol/ml as published before [12].

### Isolation and culture of PBMC

PBMC were prepared by Ficoll Hypaque (Pharmacia Fine Chemicals, Piscataway, NJ) density gradient centrifugation [13]. To assess the phenotype of T cells, PBMC were incubated in 24 well tissue culture plates (2 × 10^6^ cells/ml) in complete medium (RPMI 1640 supplemented with L-glutamine and 2% pooled human serum (PHS) and subjected to flow cytometry.

### Analysis of cell phenotype by flow cytometry

Antibodies to surface CD3 (PerCp), CD4 (FITC) and CD25 (APC) or appropriate isotype control antibodies were used in combination with antibody to intracellular Foxp3 (PE) or isotype control antibody (rat IgG2a) to identify T-reg (all antibodies were purchased from eBioscience, San Diego, CA). Cells then were fixed and acquired within 1 h of completion of staining.

To assess intracellular expression of TGFβ, PBMC were cultured with MTB H37RvL for 24 h. Monensin (1 μg/ml) was added for the final 6 hours of PBMC culture. Washed cells were labeled with antibodies to surface CD3 (PerCp), CD4 (FITC), and CD25 (APC) (all from eBioscience). Cells were fixed and permeabilized, and then stained with antibody to TGFβ (PE) (IQ Products, Groningen; The Netherlands) or isotype control antibody (IgG1 PE).

### T-cell suppression assay

PBMC (100 × 10^7^) were incubated in complete RPMI supplemented with 5% PHS in 25 cm^2^ tissue culture flasks (2.5 × 10^7^ cells/flask) in the presence of MTB H37RvL (1 μg/ml) for 5 days. At the end of incubation, CD4^+^ CD25^+^ CD127^−^ T cells were purified by immune-magnetic separation (T-reg selection kit II) (Miltenyi, Cambridge, MA). By immune-staining and FACS analysis, CD4^+^CD25^+^ CD127^−^ T cells isolated in this manner were over 90% Foxp3 positive.

A fraction of PBMC (3 × 10^7^ cells) from the same donor was kept for preparation of non-adherent responder T-cells (NAC) and monocytes (MN) by plastic adherence [14]. PBMC (5 × 10^4^ cells/well) were plated in replicate wells of 96 well round bottom tissue culture plates. NAC were harvested after 1 h and frozen in FCS10% DMSO in aliquots of 10 × 10^6^ cells/vial for use as responder cells later. Wells of adherent MN received complete RPMI and kept at 37°C. On the day of the experiment, NAC (responder cell, R) were thawed and seeded alone or in combination with MTB H37RvL expanded T-reg at several ratios of R:T-reg ( 1:3, 1:1 and 3:1) in 200 μl media/well in replicate wells of the tissue culture plates already containing adherent MN. Wells either remained un-stimulated or received anti-CD3 (0.1 μg/ml). Tissue culture plates were incubated for 4 days. On day 4 of culture, supernatants from replicate wells were aspirated and stored for assessment of IFNγ by Elisa (Pierce Endogen, Rockford, IL) later. Wells were then replenished with 200 μl of culture medium. After 18 hrs of incubation, plates were pulsed with ^3^H-Thymidine. Thymidine incorporation (^3^H-Tdr) was determined by calculating the stimulation index as follows: ^3^H-Tdr in stimulated well minus ^3^H-Tdr in un-stimulated well/^3^H-Tdr in un-stimulated well.

## Real time RT-PCR

To Quantify mRNA, Taqman methodologies using an ABI 7700 thermo cycler (Applied Biosystems, Foster City, CA) were employed. Primers and probes for ribosomal 18s (R18) RNA and Foxp3 mRNA were as before [15,16], and for IDO mRNA prepared as published [17]. Quantities of mRNA were determined by using a dilution series of target cDNA in each assay, and expression of mRNA copies were corrected to the copy numbers of R18 in the same sample.

### Inhibition of T cell Foxp3 mRNA expression by TGFβ, IL-6, or IL-8 siRNA

Adherent MN (0.2 × 10^6^) were incubated with siRNA to IL-6, IL-8, TGFβ, or control siRNA (Dharmacon Inc; Lafayette, CO) for 4 days according to methodologies provided by the manufacturer. Autologous CD4 T-cells obtained by negative selection with magnetic beads (Miltenyi), were then added to MN at a ratio of 5:1 (CD4/MN). Cultures received MTB H37RVL (1 μg/ml) or remained unstimulated for 24 hr. CD4 T-cells were then aspirated, dissolved in Tri-reagent (Molecular Science, Cincinnati, OH) and assessed for Foxp3 mRNA expression.

### Statistics

Normally distributed data sets were analyzed by student t-test. Wilcoxon or Kruskall Wallis tests were used for data sets that were not normally distributed. P value ≤ 0.05 was considered significant.

## Results

### Characterization of human T-reg expanded by MTB

**Figure 1:**
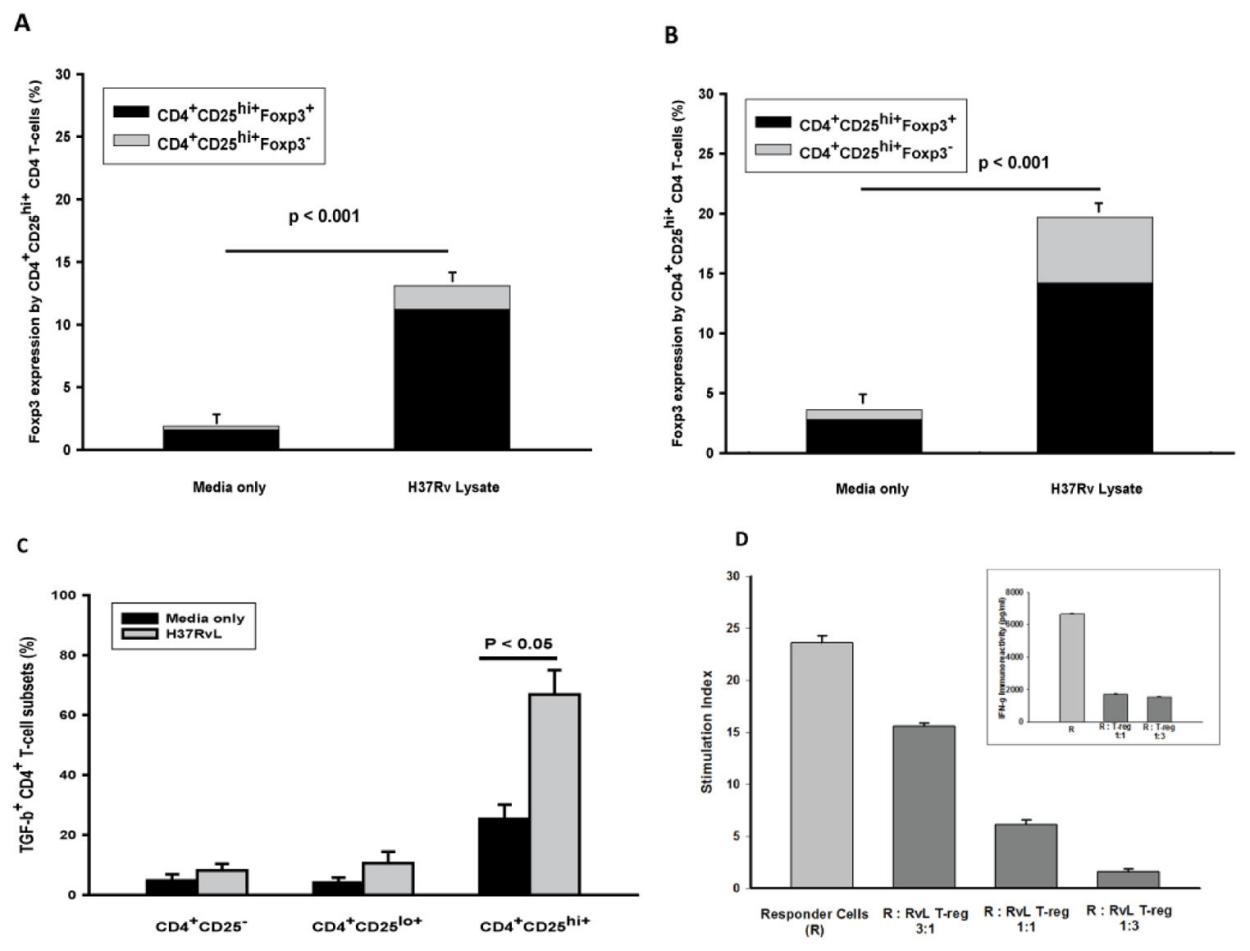
Phenotype and function of MTB H37RvL induced T-reg. PBMC from healthy TST negative and TST positive donors were cultured in media alone or media containing MTB H37RvL (1 μg/ml) for 5 days. At the end of incubation frequencies of T-reg (CD4^+^CD25^hi+^Foxp3^+^) were assessed. A. Frequencies of CD4^+^CD25^hi+^T-reg (mean ± SEM) and relative proportions of CD4^+^CD25^hi+^Foxp3^+^ T-cells in un-stimulated and MTB H37RvL stimulated cultures from TST negative subjects (n=12). B. Frequencies of CD4^+^CD25^hi+^T-reg (mean ± SEM) of and relative proportions of CD4^+^CD25^hi+^Foxp3^+^ and CD4^+^CD25^hi+^Foxp3^−^ T-cells in un-stimulated and MTB H37RvL stimulated cultures from TST positive subjects (n=6). C. Intracellular TGFβ expression of CD4^+^CD25^hi+^, CD4^+^CD25^lo+^ and CD4^+^CD25^−^ T-cells was assessed by combined intracellular and surface immune-staining following 5days of culture (n=7). Mean (± SEM) frequencies of TGFβ^+^ CD4 T-cell subsets in un-stimulated and MTB H37RvL stimulated cultures are shown. D. PBMC from TST negative subjects (n=6) were incubated with H37RvL for 5 days. Isolated CD4^+^CD25^+^ T-cells were purified and added to autologous responder (R) NAC (1 × 10^5^/well) at ratios of 3:1–1:3 (R: RvL iT-reg). Cultures were stimulated by anti-CD3 (0.1 μg/ml). IFNγ immunoreactivity was assessed in supernatants at 4 days of culture (insert). Proliferative responses were assessed by ^3^H-Thymidine incorporation on day5. Stimulation index was calculated. Data shown are expressed as mean (± SEM) of stimulation index or IFNγ activity (pg/ml).

T-regs are characterized by expression of high surface levels of CD25, intracellular expression of Foxp3, and suppression of T-cell responses to TCR stimulation [18]. To establish the phenotype of T-reg expanded by exposure to MTB, first PBMC from TST negative and TST positive subjects were cultured in presence of MTB H37RvL (1 μg/ml) and assessed for intracellular Foxp3 expression in CD4^+^CD25^hi+^ T-cells. Five day stimulation with MTB H37RvL resulted in increased CD4^+^CD25^hi+^ T-cell frequencies to 13.1 ± 0.2% (mean+SEM) in TST negative, and 19.5 ± 0.4% in TST positive donors. Intracellular expression of Foxp3 was detected in 84.6 ± 0.3% CD4^+^CD25^hi+^ T-cells in TST negative subjects (Figure 1A). By contrast, Foxp3 expression was found in 75.8 ± 0.6% of CD4^+^CD25^hi+^ T-cells from TST positive subjects (Figure 1B). To ascertain that the increase in frequencies of Foxp3^+^CD25^hi+^ CD4^+^ T-cells after stimulation by MTB in TST negative subjects was due to their expansion among CD4 T cells, rather than accentuated attrition of non-T-reg T-cells, we also assessed the absolute frequencies of CD4 T-cells after culture and found them to be comparable to time zero in TST negative subjects (data not shown). Therefore, the capacity of MTB H37RvL to induce CD4^+^CD25^hi+^ T-cells that are Foxp3^+^ is comparable between TST negative and TST positive subjects.

Next, we evaluated the intracellular expression of TGFβ in CD4^+^ T-cell subsets from TST negative subjects following culture with MTB H37RvL (1 μg/ml) for 5days. Control cultures received media alone. Frequencies of TGFβ^+^CD4^+^CD25^hi^ T-cells increased in the presence of MTB H37RvL. In a total of 7 experiments, 71.3 ± 8.1% of MTB H37RvL -stimulated as compared to 25.1 ± 4.8% of un-stimulated CD4^+^CD25^hi+^ T-cells stained positive for intracellular TGFβ at 5 days (P<0.05) (Figure 1C).

The functional profile of MTB H37RvL induced T-reg were assessed next. First, CD4^+^CD25^+^ CD127^−^T-cell were expanded in bulk by MTB H37RvL (1 μg/ml) and separated as in Methods. CD4^+^CD25^+^ CD127^−^ T cells were then co-cultured with NAC responder cells (R) at ratios of 1:3, 1:1, 3:1 R:T-reg. In control wells, responder NAC were cultured alone. T cell proliferation was assessed following stimulation with anti-CD3 (0.1 μg/ml). T cell proliferation was suppressed by addition of increasing numbers of CD4^+^CD25^+^ CD127^−^ T cells (Figure 1D). Maximal suppression was achieved when MTB H37RvL expanded CD4^+^CD25^+^ CD127^−^ T cells were added to responder NAC at a ratio of 3:1. Similarly, high amounts of IFNγ were found in supernatants of anti-CD3 stimulated NAC responder cells alone. IFNγ levels were suppressed significantly by MTB H37RvL expanded T-reg at ratios of 1:1 and 3:1 (Figure 1D insert).

Cumulatively, these data indicate that functional Foxp3 positive T reg can be induced by a preparation of MTB H37Rv components, in the absence of prior established T cell responses to MTB.

### The role of TLR ligands in MTB induced expansion of Foxp3^+^ CD4 T-cells

**Figure 2:**
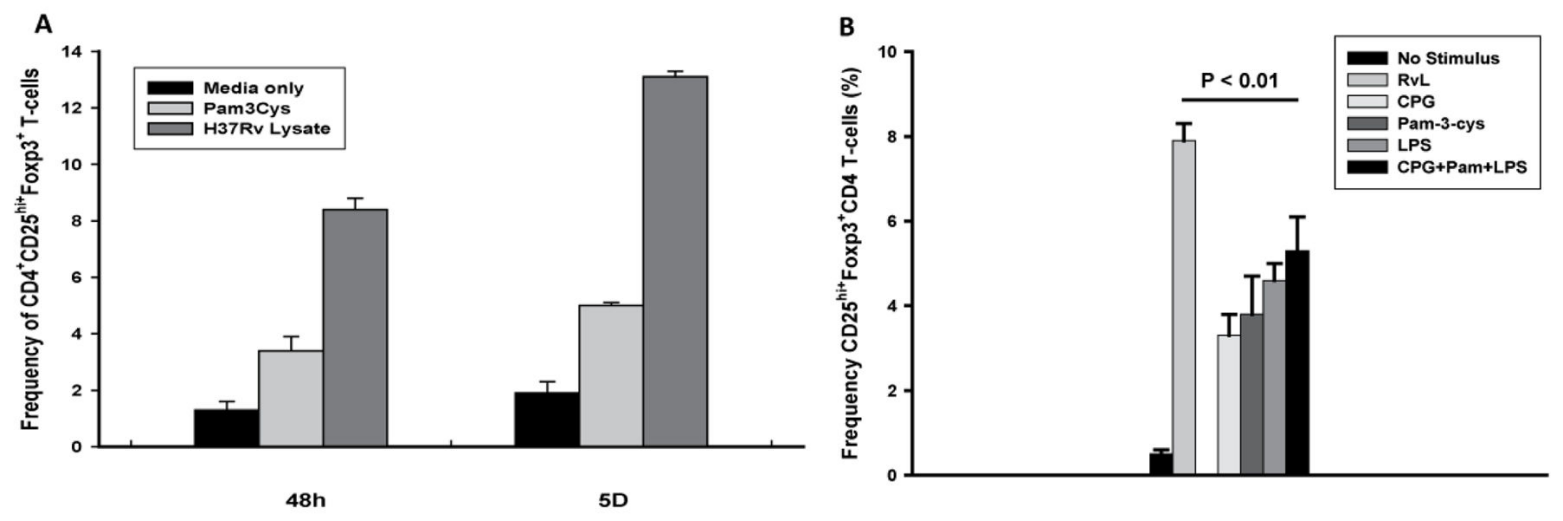
Frequencies of MTB H37RvL and TLR-ligand induced CD4^+^CD25^hi+^Foxp3^+^ T-reg. A. PBMC from TST negative subjects (n=10) were incubated with optimal amounts of MTB H37RvL (1 μg/ml), or Pam-3-cysk4 (5 μg/ml). At 48 hr and 5 days frequency of CD4+CD25hi +Foxp3+ T-cells was assessed. Induction of iTreg was significantly higher (p<0.001) by H37RvL than Pam-cysk-4. B. PBMC from TST negative subjects (n=6) were cultured with MTB H37RvL (1 μg/ml), Pam-3-cysk4 (5 μg/ml), LPS (10 ng/ml) or CPG (3 μg/ml) alone or a combination of TLR ligands (CPG + Pam cysk4 +LPS) for 5 days. Frequency of CD4^+^CD25^hi+^Foxp3^+^ T-cells was assessed Data shown are mean ± SEM of experiments.

Next the relative capacity of MTB H37RvL and the TLR-2 ligand, Pam 3-cysk4, in induction of T-reg in PBMC from TST negative subjects was assessed. Frequencies of CD4^+^CD25^hi+^Foxp3^+^ T-cells were assessed following 48 h and 5 day of culture. Both MTB H37RvL and Pam 3-cysk4 induced expansion of T-reg in a time dependent manner (Figure 2A). At 48 h, frequencies of MTB H37RvL-induced CD4^+^CD25^hi+^Foxp3^+^ T-cells exceeded that by Pam 3-cysk4 by 2.5 fold (P<0.001). Incubation for 5 days, increased frequencies of CD4^+^CD25^hi+^Foxp3^+^ T-reg further to 13.1% by MTB H37RvL, and 5.0% by Pam-3-cysk4.

Other than TLR-2 ligands, TLR-4 and TLR-9 ligands have also been found to be involved in MTB infection [19,20]. The possibility of signaling through other TLRs singly or in synergy with Pam 3-cysk4 was addressed next. PBMC were cultured with CPG, LPS, or combination of all three ligands (CPG, LPS, and Pam 3-cysk4). Cultures were maintained for 5 days (Figure 2B). The capacity of all 3 TLR ligands to support expansion of T-reg was significant as compared to un-stimulated PBMC (p<0.001, for all), however, inferior to that by MTB H37RvL. No synergism was found when all TLR ligands were used in combination.

### The role of IDO in expansion of Foxp3^+^ T cells by MTB

**Figure 3:**
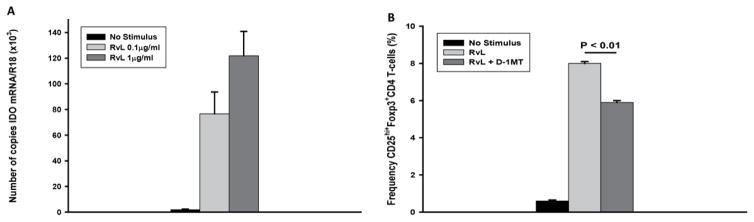
Role of MTB H37RvL in IDO mRNA induction and T-reg expansion. PBMC from TST negative subjects (n=6) were assessed for IDO expression and induction of CD4^+^CD25^hi+^Foxp3^+^ T-reg by MTB H37RvL. A. PBMC were cultured for 24 hours in the presence or absence of MTB H37RvL (0.1 or 1 μg/ml). IDO mRNA was assessed by quantitative RT-PCR in adherent MN. B. Adherent MN monolayers were prepared from PBMC of TST negative subjects, and incubated in medium alone or medium containing D-1MT (100 μmol/ml) for 2 hours. NAC were added to MN cultures at 5:1 (NAC/MN). Wells received MTB H37RvL (1 μg/ml) or media alone. After 5 days, NAC were washed and assessed for CD4^+^CD25^hi+^Foxp3^+^ by immunostaining and flow cytometry. Frequencies of MTB H37RvL-induced CD4^+^CD25^hi+^Foxp3^+^T-reg were significantly lower in cultures in which MN were pre-treated with D-1MT. Mean ± SEM of 6 experiments is shown.

To examine if support of T-reg by MTB in non-sensitized subjects involved IDO, first we examined the capacity of MTB H37RvL to induce expression of IDO mRNA in MN from TST negative subjects. For this purpose, PBMC were cultured in the presence or absence of MTB H37RvL (0.1 or 1 μg/ml) for 24 hours. Then, NAC were removed. Adherent MN were solubilized in Tri-reagent and extracted RNA assessed for IDO mRNA expression. Incubation with MTB H37RvL induced expression of IDO mRNA in MN in a dose-dependent manner (Figure 3A). Stimulation with 0.1 μg/ml and 1 μg/ml of MTB H37RvL resulted in a 35-fold and 60-fold induction of IDO mRNA (P<0.05 and P<0.01, respectively) (Figure 3A).

Next, the role of IDO in expansion of T-reg was examined using D-1MT to block IDO expression in MN. For this purpose adherent MN monolayers were prepared from PBMC of TST negative subjects, and incubated in medium alone or medium containing D-1MT (100 μmol/ml) for 2 hours. Then, NAC were added back to cultures of MN. Replicate cultures were stimulated with MTB H37RvL (1 μg/ml) or left un-stimulated for 24 h and 5 days. Adherent MN were assessed for IDO mRNA expression at 24 h. Stimulation with MTB H37RvL significantly up-regulated expression of IDO mRNA in MN (p<0.001). Pre-incubation of MN with D-1MT decreased IDO mRNA expression in MN by more than 50% (P<0.01) (data not shown). NAC collected from cultures on day 5, were assessed for T-reg (CD4^+^CD25^hi+^Foxp3^+^) expansion by immunostaining and FACS analysis. D-1MT significantly reduced expansion of CD4^+^CD25^hi+^Foxp3^+^ T-reg (P<0.01) (Figure 3B). Thus, up-regulation of IDO expression in MN in response to MTB H37RvL appears to be conducive to expansion of T-reg in PBMC from TST negative subjects.

### The role of IDO in expansion of Foxp3^+^ T cells by MTB

To examine if support of T-reg by MTB in non-sensitized subjects involved IDO, first we examined the capacity of MTB H37RvL to induce expression of IDO mRNA in MN from TST negative subjects. For this purpose, PBMC were cultured in the presence or absence of MTB H37RvL (0.1 or 1 μg/ml) for 24 hours. Then, NAC were removed. Adherent MN were solubilized in Tri-reagent and extracted RNA assessed for IDO mRNA expression. Incubation with MTB H37RvL induced expression of IDO mRNA in MN in a dose-dependent manner (Figure 3A). Stimulation with 0.1 μg/ml and 1 μg/ml of MTB H37RvL resulted in a 35-fold and 60-fold induction of IDO mRNA (P<0.05 and P<0.01, respectively) (Figure 3A).

Next, the role of IDO in expansion of T-reg was examined using D-1MT to block IDO expression in MN. For this purpose adherent MN monolayers were prepared from PBMC of TST negative subjects, and incubated in medium alone or medium containing D-1MT (100 μmol/ml) for 2 hours. Then, NAC were added back to cultures of MN. Replicate cultures were stimulated with MTB H37RvL (1 μg/ml) or left un-stimulated for 24 h and 5 days. Adherent MN were assessed for IDO mRNA expression at 24 h. Stimulation with MTB H37RvL significantly up-regulated expression of IDO mRNA in MN (p<0.001). Pre-incubation of MN with D-1MT decreased IDO mRNA expression in MN by more than 50% (P<0.01) (data not shown). NAC collected from cultures on day 5, were assessed for T-reg (CD4^+^CD25^hi+^Foxp3^+^) expansion by immunostaining and FACS analysis. D-1MT significantly reduced expansion of CD4^+^CD25^hi+^Foxp3^+^ T-reg (P<0.01) (Figure 3B). Thus, up-regulation of IDO expression in MN in response to MTB H37RvL appears to be conducive to expansion of T-reg in PBMC from TST negative subjects.

### Role of TGFβ and inflammatory cytokines in induction of Foxp3 mRNA

**Figure 4:**
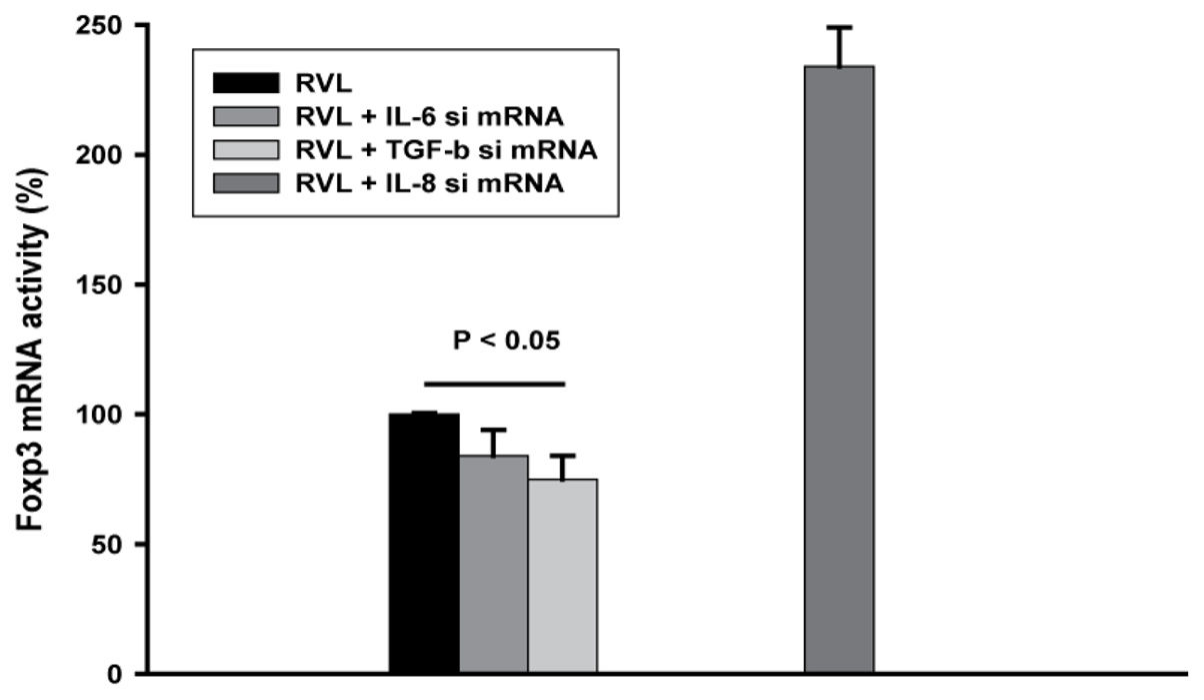
Inhibition of Foxp3 mRNA by TGFβ siRNA treatment of MN. Adherent MN were incubated with siRNA to IL-6, IL-8, TGFβ, or control siRNA for 4 days. Autologous CD4 T-cells obtained by negative selection with magnetic beads were added to MN at a ratio of 5:1; (CD4/MN). MTB H37RvL was added to some cultures. Non-adherent cells were harvested after 24 h and assessed for Foxp3 mRNA. Fold induction of Foxp3 was assessed for each condition as mRNA in MTB stimulated culture over that in control siRNA alone. Percent inhibition by IL-6, IL-8, and TGFβ siRNA compared to that by control siRNA (100%) was calculated. Mean ± SEM of 6 experiments is shown.

Next, we assessed the cytokine basis of T-reg expansion in PBMC from TST negative subjects. MTB products are potent in induction of TGFβ in MN [21,22], a cytokine well established to be involved in induction of Foxp3 expression and T-reg expansion [23,24]. However, MTB and its products induce the pro-inflammatory cytokines, IL-6 and IL-8 also [25,26]. To assess which cytokine is involved in induction of T-reg by MTB, siRNA to TGFβ, IL-6, or IL-8 were used to inhibit their expression in adherent MN. In control experiments MN were treated with control siRNA. Then, CD4 T-cells prepared from PBMC by negative immune-selection, were added to MN at a ratio of 5:1 (CD4 T-cell/MN). Induction of Foxp3 mRNA was assessed following 24 h of culture in the presence or absence of MTB H37RvL (1 μg/ml). In a total of 6 experiments, inhibition of TGFβ by siRNA reduced Foxp3 mRNA expression in CD4 T cells by a median of 25%, range 0–49% (p<0.05) (Figure 4). Interestingly, inhibition of IL-8 by siRNA increased Foxp3 mRNA expression, while siRNA to IL-6 did not affect Foxp3 mRNA expression at all.

Therefore, induction of Foxp3 mRNA expression by MTB H37RvL in T cells from TST negative subjects involves expression of TGFβ by MN.

## Discussion

Infection by MTB or exposure to its products is associated with intense microbial stimulation, through both antigenic and TLR components, and induction of a milieu that is rich in pro-inflammatory/anti-inflammatory cytokines. Here, we addressed the basis of T-reg expansion in response to MTB stimulation, in the absence of prior T cell antigen responsiveness. A simple cellular model that includes PBMC from HIV-1 un-infected TST negative subjects was developed. A crude French-press preparation of virulent MTB H37Rv, that includes all bacterial components, was used. Our findings indicate that MTB induces expansion of functional iT-reg, and that this is dependent on expression of both TGFβ and IDO by mononuclear phagocytes.

In this human *in vitro* cellular model, MTB H37RvL induced CD4^+^CD25^hi+^Foxp3^+^ T-regs that were characterized by increased intracellular expression of TGFβ, and suppression of T-cell responses. The magnitude of expansion of T-reg on day 5 of culture in response to MTB H37RvL stimulation, although higher in PBMC from TST positive individuals, was not significantly higher than in PBMC from TST negative subjects (Fig 1A and B). This contrasts with a study by Garg et al. [4], where heat-killed MTB and the MTB TLR-2 ligand, mannosylated lipoarabinomannan, resulted in expansion of T-reg in PBMC from TST positive, but not TST negative subjects. It is possible that this discrepancy is due to the fact that different stimuli and experimental set-ups were used in that study as compared to the current study. The MTB H37RvL used here, likely provides a more inclusive and diverse mix of components of virulent MTB. Others have found that, MTB infection of macaques leads to expansion of iT-reg concomitantly with development of effector T-cell responses [27]. In a murine model, Foxp3^+^ T-reg accumulated in the lung and pulmonary lymph nodes within weeks following aerosol infection with MTB [28]. In this latter study, temporary depletion of T-reg resulted in lower bacterial burdens in the lungs of treated mice as compared to control animals, implicating functionality of T-reg in suppression of control of MTB. In another study, enhanced immunogenicity of multiple vaccines, including BCG, resulted from depletion of iT-reg by concomitant administration of anti-CD25 antibody [29]. By contrast, depletion of circulating natural T-reg prior to immunization with BCG did not affect bacterial load or boost protective immune responses to BCG [30]. Differences in the profiles of natural as opposed to iT-reg, likely underlie these discrepancies. In the current study, addition of iT-reg that had been expanded by exposure of PBMC from TST negative subjects to MTB H37RvL *in vitro*, suppressed T-cell responses to anti-CD3 (Figure 1D). The potential role of iT-reg that develop in response to MTB antigens contained in vaccine candidates, in modulating establishment of protective immunity, needs to be considered.

Another aspect of the current study was to examine the contribution of signaling through TLRs in T-reg expansion in MTB naïve individuals. Evidence for a role for TLR-2, TLR-4 and TLR-9 in T-reg expansion has been shown [5,31–35]. MTB lipoproteins, such as 19kD (Lpqv) antigen and LprG [5,34] or ManLAM [36,37], are all TLR-2 ligands and involved with development of immune responses. Engagement of TLR-2 in the absence of antigen presenting cells was sufficient to induce proliferation of T-reg [6]. Here, TLR2 ligand even in combination with a cocktail of TLR-4- and TLR-9 ligands were inferior to MTB H37RvL in expansion of iT-reg (Figure 2), may imply non-TLR mechanisms in their expansion. Whether TLR2 ligation leads to reduction in the suppressive function of iT-reg as shown before [7] was not addressed here.

Molecules such as IDO, which are induced in DC and mononuclear phagocytes, have been shown to be key in T-reg expansion [33,38–40]. Interestingly, IDO-mediated auto-regulatory loops supporting T-reg expansion appear to involve TGFβ [41]. Our data show that MTB H37RvL is a potent inducer of IDO expression in monocytes, and that IDO expression plays a role in expansion of T-reg, as co-culture with its inhibitor D-1MT [12] not only inhibited expression of IDO mRNA in MN, but also decreased frequencies of T-reg (Figures 3A and 3B). The effect of inhibition of IDO by D-IMT- in expansion of iT-reg was partial, implicating effect of other molecules also. IDO has been shown to activate T reg induction and block their conversion to TH17-like T cells [10]. Whether this mechanism is also involved in expansion of iT-reg in response to MTB needs to be examined.

Among molecules implicated in expansion of iT-reg in response to MTB, the cytokine TGFβ features prominently [23]. High amounts of TGFβ are induced in mononuclear phagocytes in response to MTB and its protein and non-protein products [21,22,42]. Here, inhibition of MTB-induced TGFβ mRNA expression by siRNA in MN, reduced expression of Foxp3 mRNA in CD4 T-cells (Figure 4). However, intracellular expression of TGFβ characterized CD4^+^CD25^hi^ T-cells induced by H37RvL in PBMC from TST negative subjects *in vitro* (Figure 1C). This observation may indicate that an auto-regulatory circuit perpetuating T-reg expansion involving TGFβ may be established by MTB. Thus, TGFβ produced by mononuclear phagocytes in response to MTB H37Rv and its components support expansion of TGFβ producing T-reg. TGFβ expressed by iT-reg further supports expansion of iT-reg. The finding here of increased Foxp3 mRNA expression by inhibition of IL-8 expression by siRNA treatment of MN was unexpected, however, may indicate a possible role for IL-8 in modulation of T-reg expansion. Interestingly, T-reg among pleural mononuclear cells from TB patients were found to be IL-8 reactive [8]. Collectively, these data suggest auto-regulatory circuits, both positive (by TGFβ) and negative (by IL-8) regulation of Foxp3 mRNA expression in iT-reg.

In summary, data presented here make a strong argument for a role for MTB and its components in expansion of iT-reg that are suppressive of T cell responses in human mononuclear cells from MTB non-sensitized subjects. Further, these data suggest a role for mononuclear phagocyte TGFβ and IDO expression in induction of MTB-induced iT-reg in TST negative subjects. Whereas iT-reg thus expanded may ultimately be controlled by other components of innate immunity, is not known. However, activated NK cells have been shown to lyse MTB-induced iTreg through NKG2D [43]. Whether NK cells from peripheral circulation are recruited efficiently to sites of exposure to MTB antigens is unknown. Given the potential interference to development of protective immune T-cell responses to novel MTB antigens by iT-reg, implications in the design of future vaccines against MTB needs to be considered.
